# Supplementary material for: Proteasomes accumulate in the plant apoplast where they participate in microbe-associated molecular pattern (MAMP)-triggered pathogen defense
Source: Nat Commun. 2025 Feb 14;16:1634. doi: 10.1038/s41467-025-56594-3 (PMC11829042; doi:10.1038/s41467-025-56594-3)
Supplement: Supplementary file 1 — Supplementary Information [file 41467_2025_56594_MOESM1_ESM.pdf]

Supplementary Tables/Figures/Method

Supplementary Table 1 and Supplementary Figures 1-9

Supplementary Method – Synthesis of Syringolin A.

Proteasomes Accumulate in the Plant Apoplast Where They Participate in Microbe-Associated Molecular Pattern (MAMP)-Triggered Pathogen Defense

Hana Zand Karimi, Kuo-En Chen, Marilee Karinshak, Xilin Gu, Jason K. Sello, and Richard D. Vierstra

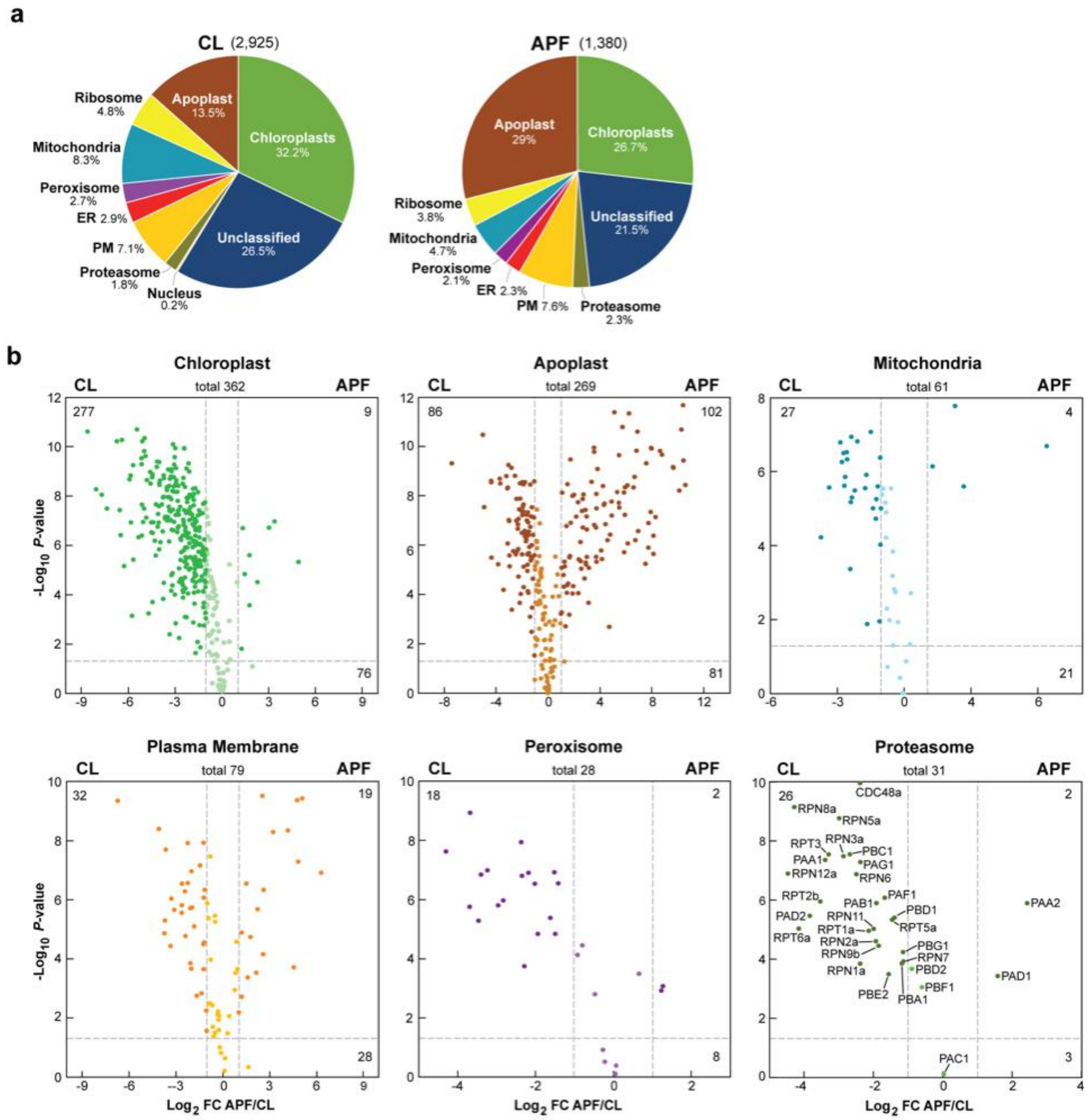

Supplementary Figure 1 | Proteomic analysis of the *Arabidopsis* leaf APF showing enrichment of specific compartments/protein complexes. Clarified CL and the total APF were digested with trypsin and subjected to LS-MS/MS. Proteins present in the *Arabidopsis* proteome database were identified by Proteome Discover 2.5 whose abundances were then determined semi-quantitatively by (**a**) the number of proteins detected or (**b**) by combined ion intensities from the MS1 scans (e-values). The CL and APF were assayed by four biological replicates each analyzed by two technical replicates. Only those proteins detected in at least one technical replicate for all four biological replicates were included for each genotype. All identified proteins were included in the analyses. **a,** Pie chart showing the percent of proteins assigned to specific compartments/protein complexes based on the number of proteins detected.

Assignments were either determined in GO or by a master list of apoplastic proteins (see **Supplemental data 1-3**). **b,** Volcano plots comparing the abundances of proteins assigned to specific cellular compartments and proteasomes in the CL and APF. Relative abundances in the APF versus CL were based on MS1 ion intensities of each protein after normalization based on the combined MS1 ion intensities for the entire sample. The total numbers assigned to each compartment are indicated. Those proteins with significant enrichment in the CL versus APF fractions (Log_2 FC ≥1 or ≤-1 and *P*-value<0.05) are highlighted by the darker color circles. The numbers of proteins with significant or insignificant differences in abundance between CL and APF are indicated in the corners of the graphs. The dashed lines indicate the significance boundaries based on both (Log_2 FC ≥1 or ≤-1 and *P*-value<0.05). Subunits of the *Arabidopsis* proteasome (both CP and RP) and CDC48 are indicated.

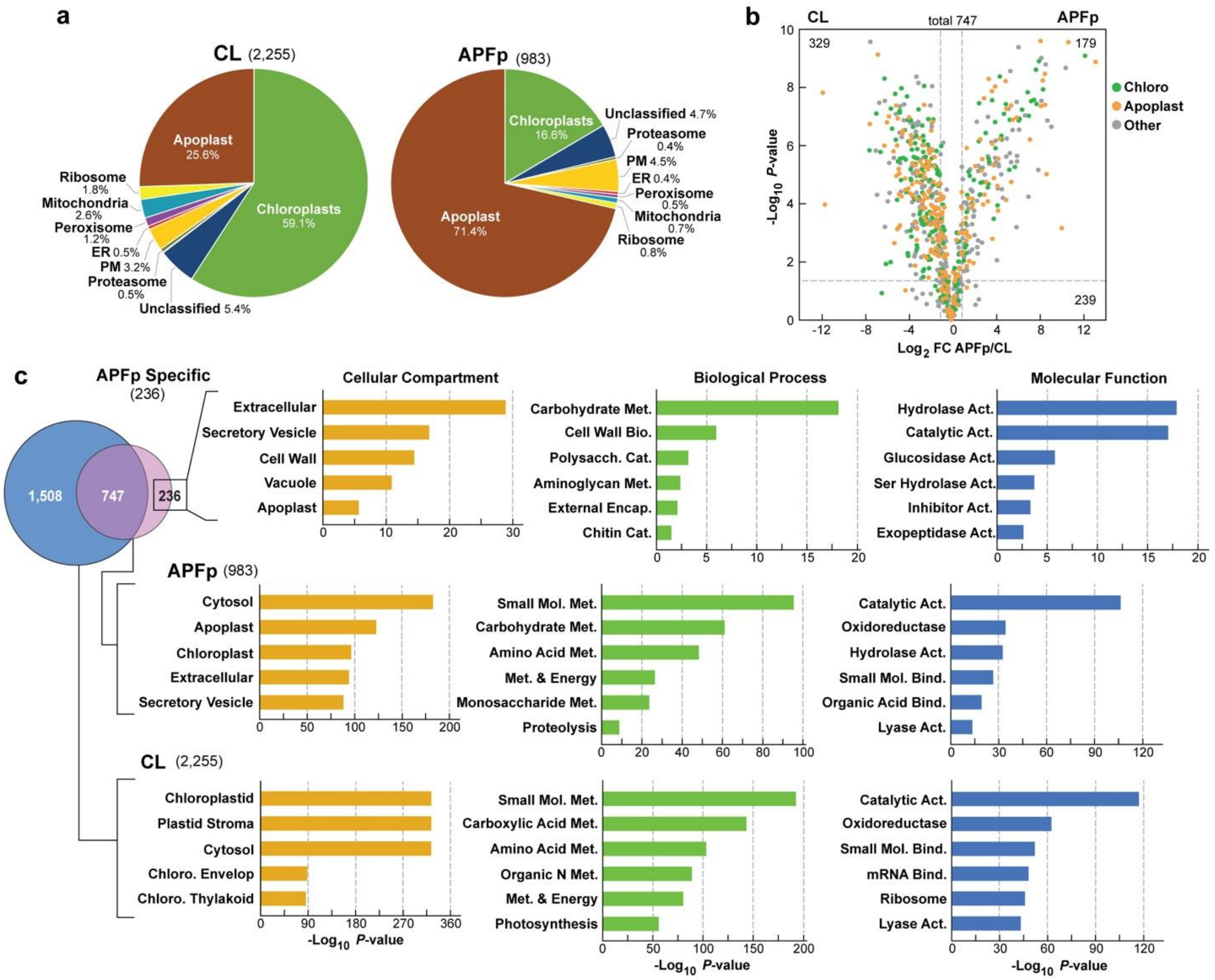

Supplementary Figure 2 | Proteomic analysis of the *Arabidopsis* leaf APFp.
Total protein from the clarified CL and the APF pellet (APFp) were digested with trypsin and subjected to LS-MS/MS. Proteins present in the *Arabidopsis* proteome database were identified by Proteome Discover whose abundances were then determined semi-quantitatively based on the combined ion intensities determined from the MS1-scans (e-values). The CL and APFp were each assayed by four biological replicates each measured with two technical replicates; protein abundances were then normalized based on the total MS1 ion intensities for all proteins in each. Only those proteins detected in at least one technical replicate for each of the four biological replicates were included. Values for the Rubisco large and small subunits were removed from the APFp prior to analysis to avoid their abundance bias. **a,** Pie charts showing the percentage of proteins localized to specific compartments/complexes in the APFp versus CL (983 and 2,255, respectively) using assignments either provided by GO or from a master list of apoplastic proteins (See **Supplementary Data 6-8**). ER, endoplasmic reticulum. PM, plasma membrane. **b,** Volcano plots of 749 proteins found in both the CL and APFp showing their relative abundances based on their MS1 ion intensities. Green and orange points identify chloroplast and apoplast proteins, respectively. All others are colored in grey. The numbers of proteins with significant or insignificant differences in abundance between

CL and APFp are indicated in the corners of the graphs. The dashed lines indicate the significance boundaries based on both Log_2 FC $\geq$1 or $\geq$-1 and *P*-value of significance <0.05. **c,** Functional analysis of the CL (2,255 total), APFp (983 total), and APFp-specific proteins (236 total) as determined by GO. Shown are the top 5-6 GO terms for each set based on the Cellular Compartment, Biological Process, and Molecular Function categories. Top left, Venn diagram showing the overlap of the fractions.

Supplementary Table 1.
Top Apoplastic Proteins in the Arabidopsis Leaf APFp[a]

Chromosome Location	Protein Name	Function/Activity	Location[b]	Rank[c]	Def.[d]
At1g75040	PR5	Pathogenesis Related-5	Apo	1	+
At2g10940		bifunctional inhibitor/lipid transfer	Apo	2	
At5g13980		α-mannosidase (family 38)	Apo	3	
At1g09750		aspartyl protease	Apo	4	
At3g57260	BGL2/PR2	β-1-3 glucanase-2	Apo	5	+
At1g78830	MNB1	Curulin (mannose-binding) lectin	Apo/Golgi	6	
At5g26000	BGLU38/TGG1	thioglucoside glucohydrolase-1	Apo/Chlo	7	+
At5g25980	BGLU37/TGG2	thioglucoside glucohydrolase-2	Apo/Chlo	8	+
At1g21670	DPP6	GSDL-like lipase	Apo/CW	9	+
At3g08030		cell wall DUF642	CW	10	
At3g16670	OLE1	extensin family	Apo	11	
At4g23170	CRK9/EP1	Cys-rich receptor kinase-9	Apo/PM	13	+
At5g08380	GAL1	α-galactosidase-1	Apo/CW	14	+
At4g20840	BBE21	oligogalacturonide oxidase-2	Apo	15	+
At5g64570	XYL4	β-D-xylosidase (family 3)	Apo/CW	16	+
At1g76160	SKS5	oxidoreductase	Apo	17	+
At5g67360	SBT1.7	subtilisin-like Ser protease	Apo	18	+
At3g52840	βGAL2	β-galactosidase-2	Apo	20	+
At1g29670	GDSL1	GDSL acetyltransferase/lipase	Apo	22	
At3g14210	EMS1	glucosinolate hydrolase	Apo	24	+
At2g28470	βGAL8	β-galactosidase-8	Apo/CW	25	
At3g18490	ASPG1	aspartyl protease	Apo	27	+
At2g28000	CPN60A	chaperonin-60α	Apo/Chlo	29	
At4g20830	OGOX1	oligogalacturonide oxidase	Apo	30	+
At2g38540	LTP1	lipid transfer protein-1	Apo/CW	32	
At2g14610	PR1	Pathogenesis Related-1	Apo/CW	33	+
At3g55440	TPI1	triosephophate isomerase	Apo/Chlo	36	
At3g14420	GOX1	glycolate oxidase-1	Apo/Per	39	+
At3g05730	DEFL205	defensin-like protein-205	Apo	40	+
At1g55490	CPN60B	chaperonin-60α	Apo/Chlo	42	+
At5g10560	BXL6	β-xylosidase-6	Apo/Cyto	43	+
At1g78850	GAL1	Curculin-like mannose-binding lectin-1	Apo	45	+
At3g04120	GAPC	glyceraldehyde 3-P dehydrogenase	Apo/Cyto	47	+
At3g16530		chitin-induced lectin binding	Apo/Cyto	49	+
At3g57240	BG3	β-1-2-glucanase-3 (family-17)	Apo	51	+
At5g10760	AED1	apoplastic aspartyl protease	Apo	52	+
At2g43570	CHI	chitinase	Apo	54	+
At5g11720	AGLU1	α-glucosidase (family 31)	Apo	55	
At1g79720		aspartyl protease	Apo	56	
At4g02520	GST2	glutathionine S-transferase-2	Apo/Cyto	59	
At5g63800	βGAL6	β-galactosidase-6	Apo	60	+
At4g27520	NODL2	early nodulin-like-2	Apo	62	
At4g12910	SCPL20	Ser-carboxypeptidase-like-20	Apo	64	
At4g23670		polyketide cyclase/dehydratase	Apo/Cyto	66	
At4g34260	AXY8	α-L-fucosidase	Apo	67	+
At4g16500	CYS4	Cystatin	Apo	69	+
At3g10740	ARAF1	α-L-arabinofuranosidase-1	Apo	71	+
At4g12880	NODL19	early nodulin-like-19	Apo	73	+
At3g45970	EXPL1	expansin-like-L1	Apo/CW	75	
At1g65930	CICDH	NADP-dep. isocitrate dehydrogenase	Apo/CW	79	+

[a] Top 50 ranked apoplast proteins identified in the APFp based on MS1 ion counts as determined by LC-MS/MS.

[b] Most likely location(s) as defined in TAIR. Apo, apoplast. Chlo, chloroplast. CW, cell wall. Cyto, cytosol. Per, peroxisome. PM, plasma membrane.

[c] Abundance rank based on MS1 ion counts after removing Rubisco large and small subunits.

[d] +, Linked previously to biotic defense.

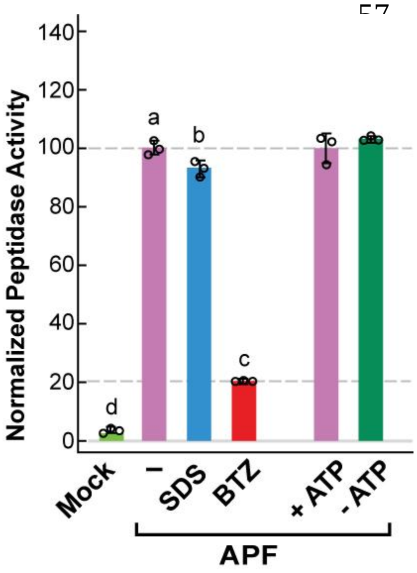

Supplementary Figure 3 | Sensitivity of ex-proteasome activity to SDS and ATP. Proteasome activity was determined in the APF using the fluorogenic substrate Suc-LLVY-AMC assayed at 37°C. Regents tested include 0.02% SDS, 50 µM BTZ, or 1 mM ATP, which were added to the APF 5 min before assay. Mock, reaction without APF. Bars reflect the mean (±SD) of three technical replicates. Different letters above the bars indicate a significant difference from others using a one-way ANOVA followed by Tukey's test to determine significance. Individual data points are included.

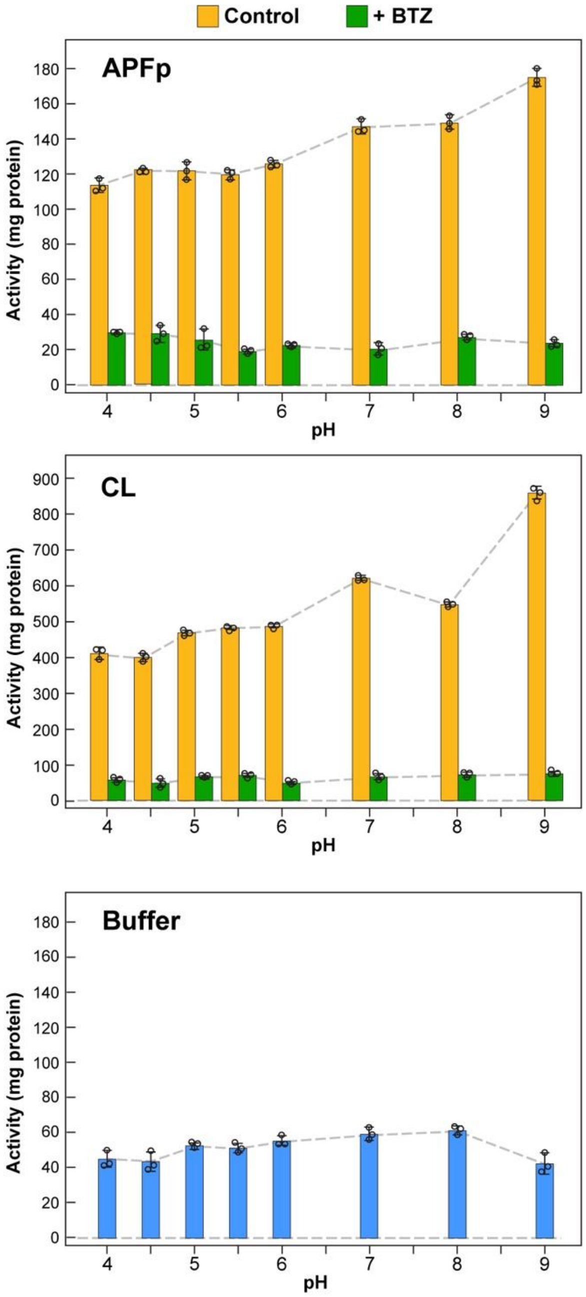

Supplementary Figure 4 | Proteasomes from both the CL and the APF are catalytically activity over a wide range of pHs. The CL and APF fractions were mixed with a broad pH range buffer adjusted to the indicated pHs and assayed for activity using the fluorogenic substrate Suc-LLVY-AMC at 37°C with or without 50 µM BTZ. Shown are fluorescence measurements for the APF, the CL, and the broad pH range buffer alone as each pH. Bars reflect the mean (±SD) of three technical replicates. Individual data points are included.

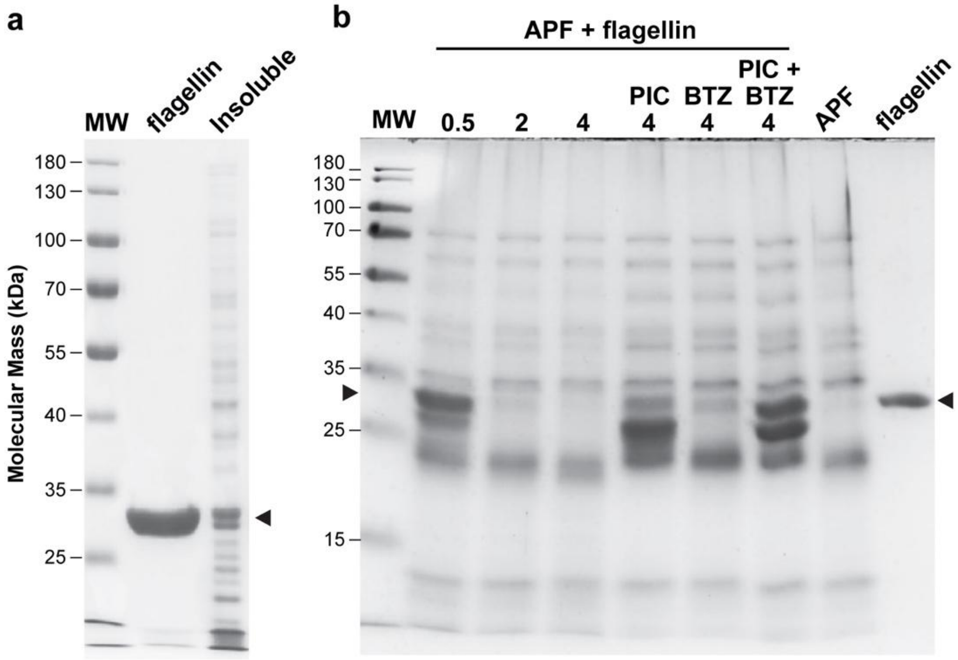

Supplementary Figure 5. Purification and proteolytic sensitivity of *P. syringae* flagellin. a, Purification of flagellin from *P. syringae* pv *tomato* DC3000. The lanes show the purified 31-kDa protein and the insoluble material remaining after final acid denaturation step. **b,** Sensitivity of flagellin to proteases within the *Arabidopsis* APF. Flagellin (4 µg) was incubated at room temperature with 400 µL of APF with or without a 1X concentration of PIC or 50 µM BTZ, and quenched at the indicated times with SDS-PAGE sample buffer. APF and purified flagellin alone are shown in the two right lanes. Arrowheads located flagellin. MW, molecular mass markers. Samples in both panels were subjected to SDS-PAGE and stained for protein with Coomassie Blue.

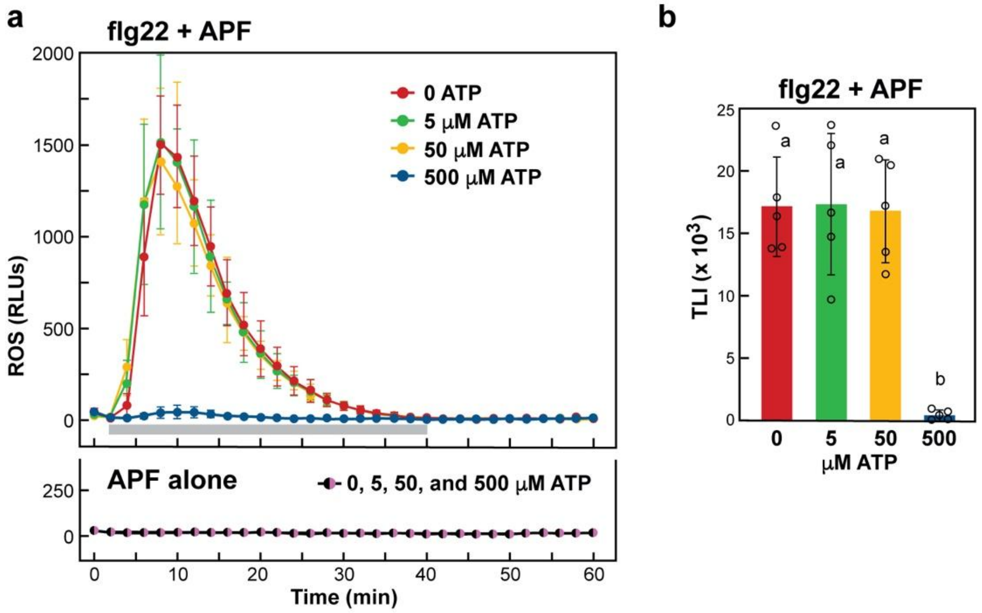

Supplementary Figure 6 | Low concentrations of ATP does not inhibit the ROS burst elicited by the APF mixed with flg22. *Arabidopsis* leaf discs floating on a luminol and horseradish peroxidase solution were incubated with APF with or without 100 nM of flg22 and increasing concentrations of ATP, and immediately assayed for ROS production every 2 min by relative fluorescence units (RLUs). **a,** time course for fluorescence output with APF alone (lower panel) or the APF containing flg22 (upper panel). Each point represents the mean of five biological replicates (±SD). Horizontal grey bar indicates the measurement timeframe for determining the total luminescence intensity (TLI). **b**, TLI of the ROS burst comparing leaf discs treated with APF, flg22, and increasing concentrations of ATP. Each bar shows the mean TLI of five biological replicates starting at min 2 to 40 after flg22 addition. TLI values were calculated by subtracting the RLU values generated with APF and 0 μM ATP alone and integrating the adjusted RLU values to generate the TLI. The bars represent the mean TLI (±SD). The individual data points are included. Different letters above the bars indicate a significant difference from others using a one-way ANOVA followed by Tukey's test to determine significance.

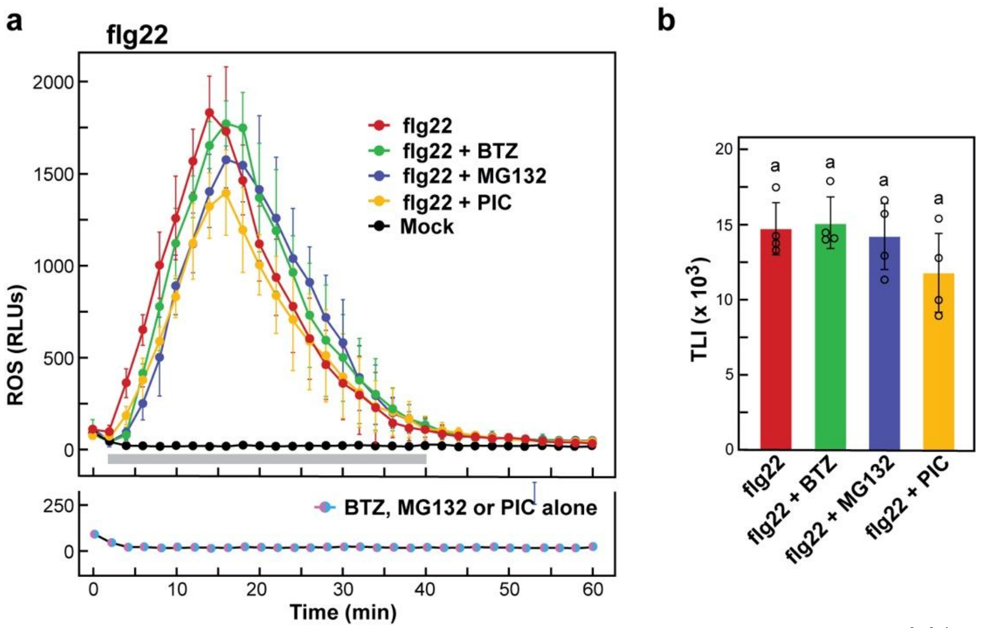

Supplementary Figure 7 | Proteasome and protease inhibitors do not inhibit ROS bursts elicited by flg22. *Arabidopsis* leaf discs floating on a luminol and horseradish peroxidase solution were incubated with 100 nM of flg22 or an equivalent volume of DMSO (Mock) and immediately assayed for ROS production every 2 min by relative fluorescence units (RLUs). **a,** time course for fluorescence output of leaf discs with or without 62.5 µM of the proteasome inhibitors BTZ and MG132, or a 1X concentration of PIC that inhibits a collection of peptidase/proteases. The lower graph in each panel shows the ROS burst time course for leaf discs treated with inhibitors alone. Each point represents the mean of four biological replicates (±SD). **b**, Total luminescence intensity (TLI) of the ROS burst comparing leaf discs treated with flg22 alone or with the inhibitors. Each bar shows the mean TLI of four biological replicates generated by flg22 in the presence or absence of inhibitors starting at min 2 to 40 after flg22 addition. TLI values were calculated by subtracting the RLU values generated with the Mock treatment from those generated with flagellin at each time point and integrating the adjusted RLU values to generate the TLI. The bars represent the mean TLI (±SD). The individual data points are included. Different letters above the bars indicate a significant difference from others using a one-way ANOVA followed by Tukey's test to determine significance. Mock, ROS measured with water alone.

155

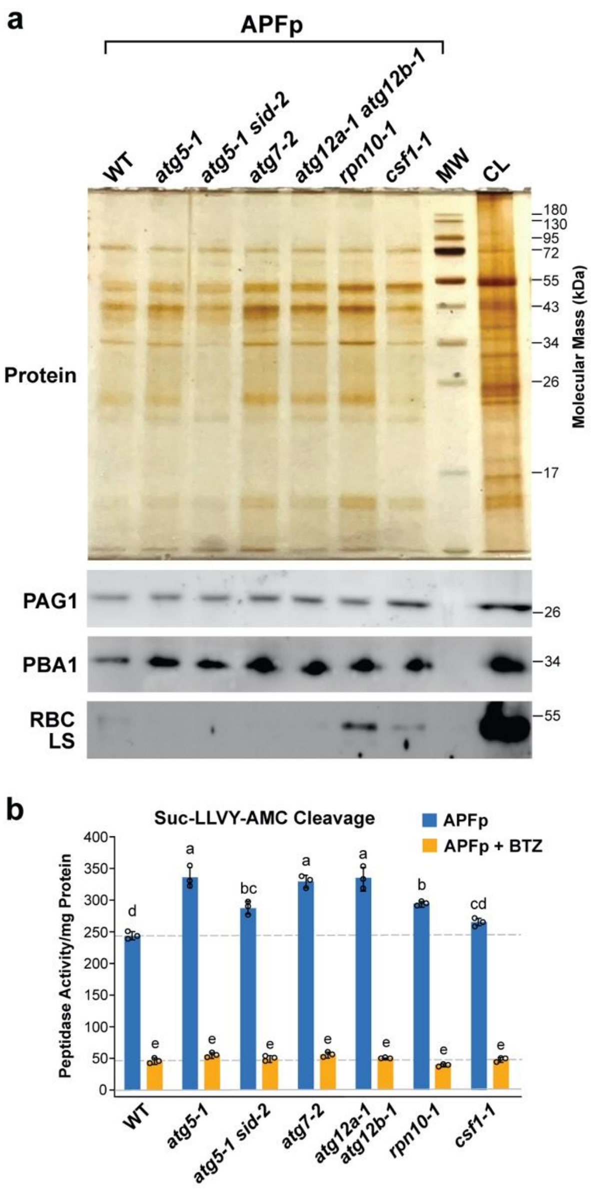

Supplementary Figure 8 | Mutants affecting autophagy and amphisome assembly do not affect ex-proteasome accumulation. APFp was isolated from *Arabidopsis* leaves either wild type (WT) or homozygous for mutants either blocking autophagy (*atg5-1*, *atg7-2*, and *atg12a-1 atg12b-2*), the selective autophagic clearance of proteasomes (*rpn10-1*), or amphisome assembly (*csf-1*). Also included was a double *atg5-1 sid-2* mutant that suppresses the effects of salicylic acid on autophagy. **a,** SDS-PAGE and immunoblot analysis of the APFp. Top panel shows the protein profile by SDS-PAGE as detected by silver staining. Lower panels show immunoblots of the fractions using antibodies against the PAG1 (α7) and PBA1 (β1) subunits of the CP, and the Rubisco large subunit. The CL was included for comparison. **b,** Proteolytic activity of proteasomes in the APFp samples using the fluorescent Suc-LLVY-AMC substrate with or without 50 µM BTZ. Each bar represents the mean (±SD) of three biological replicates. Different letters above the bars indicate a significant difference from others using a one-way ANOVA followed by Tukey's test to determine significance. Individual data points are included.

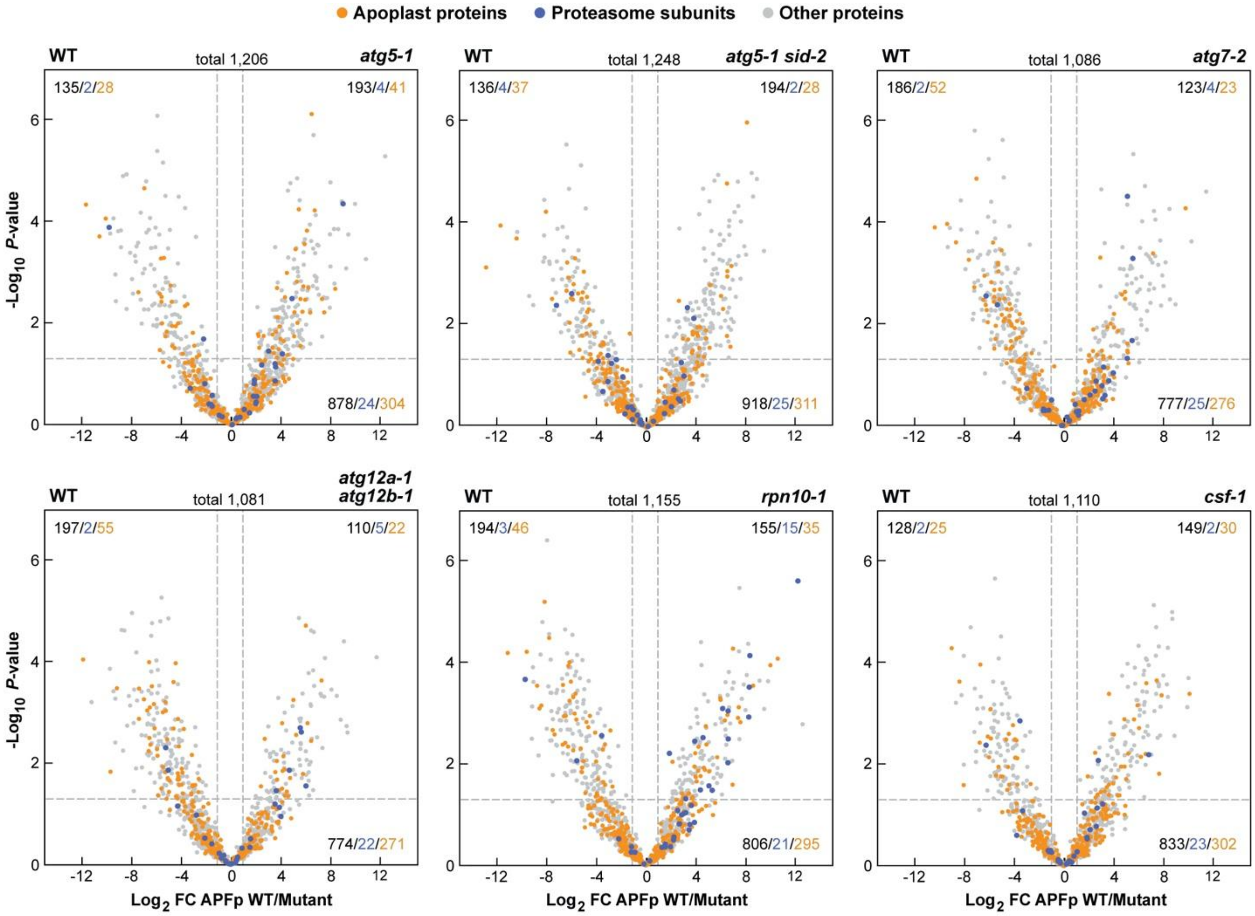

Supplementary Figure 9 | Mutants affecting autophagy and amphisome assembly minimally affect the profile of APFp proteins and the accumulation of ex-proteasomes. Shown are volcano plots comparing LC-MS/MS data from the APFp isolated from *Arabidopsis* leaves either wild type (WT) or homozygous for mutants blocking autophagy (*atg5-1*, *atg7-2*, and *atg12a-1 atg12b-2*), the selective autophagic clearance of proteasomes (*rpn10-1*), or amphisome assembly (*csf-1*). Also included was a double *atg5-1 sid-2* mutant that blocks the effects of salicylic acid on autophagy. The APFp samples were trypsinized and analyzed by LC-MS/MS. Each genotype was assayed by four biological replicates each analyzed by two technical replicates. Only those proteins detected in at least one technical replicate for all four biological replicates were included for each genotype. The abundance of each protein was then compared after normalization of each sample based on the combined MS1 ion intensities (e-values) of each. Proteasome subunits and proteins enriched in the apoplast are colored in blue and orange, respectively. All other proteins are shown in grey. Values for the Rubisco large and small subunits were removed from the lists prior to analysis. The total protein numbers in common between samples are indicated at the top of each panel. The dashed lines delineate those AFPp proteins with specific enrichment in WT or mutant (Log_2 FC ≥ 1 or ≤ -1 and *P*-value<0.05).

Supporting Information for the Chemical Synthesis of Syringolin A

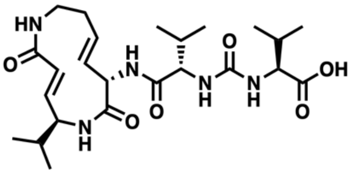

Syringolin A

Xilin Gu, Ph.D. and Jason K. Sello, Ph.D. Department of Pharmaceutical Chemistry, University of California, San Francisco, Genentech Hall, 600 16th Street, San Francisco, CA 94158

General information

All reactions were carried out in oven-dried glassware. TLC was performed using precoated silica gel 60 plates and visualized by UV fluorescence quenching. Flash chromatography was performed using ZEOprep 60 silica gel (pore diameter 6 nm, particle size 40-63 μm). Yields refer to chromatographically pure compounds. ^{1}H NMR spectra were recorded on a Bruker Avance III HD 400 MHz spectrometer or Bruker Avance 600 MHz unless otherwise noted. All signals are reported in ppm with the internal reference of 7.26 ppm for chloroform, 2.50 ppm for DMSO, 3.30 ppm for methanol, 7.16 ppm for benzene as standard. Data are presented as follows: multiplicity (s = singlet, d = doublet, t = triplet, m = multiplet, br = broad, ABq = AB quartet, dd = doublet of doublet, dt = doublet or triplet), coupling constant (J/Hz) and integration.

Reagents

The Garner's aldehyde (**1**) was prepared according to the reported procedure.[1] Valinol, PyAop and DIPEA were purchased from ChemImpex International. Isocyanate **11** was purchased from TCI America. All other reagents were purchased from Sigma-Aldrich.

Overview of the Syringolin A Synthetic Scheme

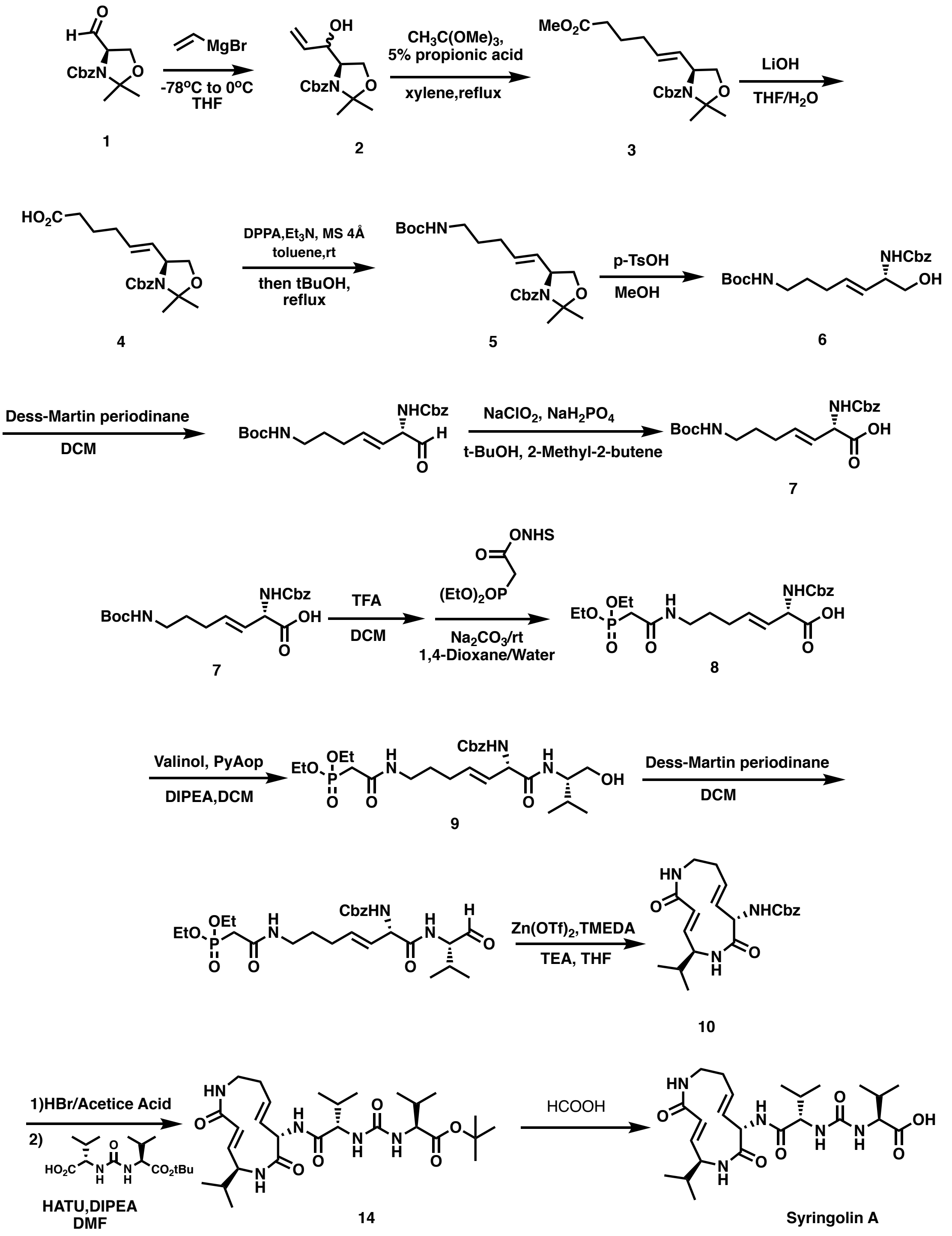

The synthetic scheme is based on routes published by Stephenson[1] and Pirrung[2].

Procedures and Spectral Data

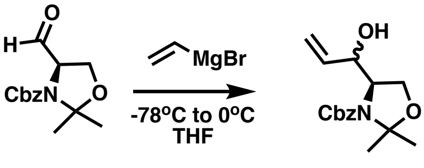

1 2

A solution of Garner aldehyde **1** (10.52 g, 40.0 mmol, 1.0 equiv) in dry THF (100 mL) was cooled to -78 °C and treated with 1 M vinylmagnesium bromide in THF (120 ml, 120.0 mmol, 3.0 equiv) under argon atmosphere. The temperature was allowed to rise to -15°C during 2h. After quenching with saturated NH_4Cl solution (80 mL), the reaction mixture was extracted with Et_2O (200 mL × 3). The combined organic layers were washed with brine, dried (Na_2SO_4), filtered and concentrated. The resulting crude product was purified by column chromatography (25% ethyl acetate in hexane) to afford **2** as a colorless oil (9 g, 30.89 mmol, 77%).

Rf (1:3 EtOAc/hexane): 0.19

^1H NMR (600 MHz, benzene-d6, 75 ℃):δ 7.04-7.23 (m, 5 H), 5.68-5.83 (m, 1 H), 5.22 (dd, J = 44.8, 17.2 Hz, 1 H), 4.96-5.10 (m, 1 H), 5.03 (s, 2 H), 4.30-4.38 (m, 1 H), 3.84-3.96 (m, 1 H), 3.76-3.84 (m, 1 H), 3.60 (dd, J = 15.8, 8.8 Hz, 1 H), 1.62 (s, 3 H), 1.43 (s, 3 H).

The ^1H spectral data match the literature precedent.[2]

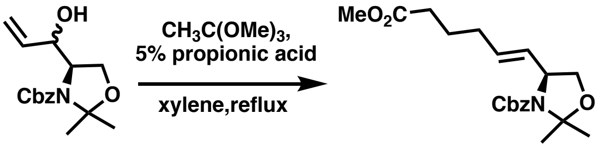

2 3

Vinyl alcohol **2** (9g, 30.89 mmol, 1.0 equiv) was dissolved in xylene (50 mL) followed by addition of trimethyl orthoacetate (31.1 mL, 246.4 mol, 8.0 equiv) and propionic acid (0.042 mL). The reaction mixture was heated to reflux under argon atmosphere and stirred for 24h before evaporation to dryness. The resulting crude product was purified by column chromatography using gradient elution (10% to 25% ethyl acetate in hexanes) to afford compound **3** as a colorless oil (6.2 g, 17.15 mmol, 55%).

Rf (1:4 EtOAc/hexanes): 0.30

^1H NMR (600 MHz, benzene-d6, 75 ℃)δ 7.05-7.25 (m, 5 H), 5.35-5.45 (m, 2 H), 5.09 (ABq, J = 12.2 Hz, Δν= 35.6 Hz, 2 H), 4.1-4.2 (m, 1 H), 3.67 (dd, J = 8.8, 6.2 Hz, 1 H), 3.46 (dd, J = 9.0, 2.2 Hz, 1 H), 3.36 (s, 3 H), 2.16 (t, J = 5.4 Hz, 2 H), 2.05-2.11 (m, 2 H), 1.70 (s, 3 H), 1.54 (s, 3 H).

The ^1H spectral data match the literature precedent.[2]

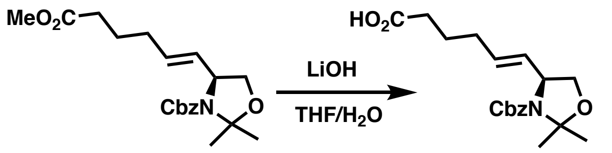

3 4

To a solution of methyl ester **3** (4.58 g, 13.2 mmol, 1.0 equiv) in THF-MeOH-H_2O (3:1:1) (50 mL) was added LiOH·H_2O (2.16 g, 52.8 mmol, 4.0 equiv) at 0˚C. The heterogeneous reaction mixture was then warmed to room temperature and stirred overnight. The resulting solution was diluted with water (100 mL) and washed with diethyl ether(100 mL x 2). 1 M HCl solution was carefully added to the resulting aqueous layer until pH = 3.0. The aqueous layer was extracted with ethyl acetate (3 × 50 mL) and combined organic layers were washed with brine, dried (Na_2SO_4), filtered and concentrated. The resulting crude product was recrystallized with ethyl acetate and hexanes (1:6, 70 mL) to afford compound **4** as a colorless solid (3.36g, 12.7 mmol, 73%).

^{1}H NMR (600 MHz, benzene-d6, 75 °C): δ 7.06-7.24 (m, 5 H), 5.36-5.48 (m, 2 H), 5.09 (ABq, J = 12.5 Hz, $\Delta\nu$= 60.0 Hz, 2 H), 4.10-4.19 (m, 1 H), 3.69 (dd, J = 8.8, 6.0 Hz, 1 H), 3.47 (dd, J = 8.8, 2.2 Hz, 1 H), 2.11 (t, J = 6.0 Hz, 1 H), 2.06 (m, 1 H), 1.70 (s, 3 H), 1.54 (s, 3 H).

The ^{1}H spectral data match the literature precedent.[2]

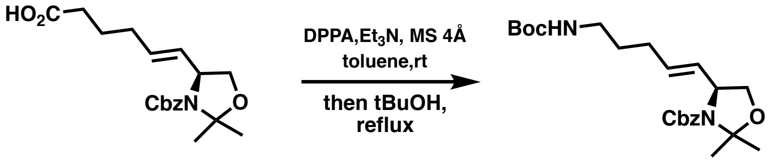

4 5

A flame-dried two-necked round-bottom flask fitted with a reflux condenser was charged with acid **4** (3.2 g, 9.60 mmol, 1.0 equiv), triethylamine (1.32 mL, 9.60 mmol, 1.0 equiv), 4 Å molecular sieves (10.0 g) and toluene (100 mL). To this solution was added diphenylphosphoryl azide (2.92 g, 10.56 mmol, 1.1 equiv) dropwise at 0˚C under nitrogen atmosphere. The reaction mixture was then warmed to room temperature and stirred for 2 h. A solution of tert-butanol (9.18 mL, 94.0 mmol, 10.0 equiv) and triethylamine (1.32 mL, 9.60 mmol, 1.0 equiv) in toluene (50 mL) was added slowly. The reaction mixture was then heated to reflux and stirred overnight. The solvent was removed under reduced pressure and the residue was purified by column chromatography using gradient elution (10% to 25% ethyl acetate in hexanes) to afford compound **5** as a colorless oil (2.92 g, 6.98 mmol, 73%).

Rf (1:3 EtOAc/hexanes): 0.25

^{1}H NMR (600 MHz, benzene-d6, 75 ℃):δ 7.07-7.25 (m, 5 H), 5.3-5.4 (m, 2 H), 5.11 (ABq, J = 7.5 Hz, $\Delta\nu$= 57.5 Hz, 2 H),4.08-4.19 (m, 2 H), 3.70 (m, 1 H), 3.47 (m, 1 H), 2.95-3.03 (m, 1 H), 2.86-2.93 (m, 1 H), 1.38-1.65 (m, 2 H), 1.68 (s, 3 H), 1.53 (s, 3 H), 1.45 (s, 9 H).

The ^{1}H spectral data match the literature precedent.[2]

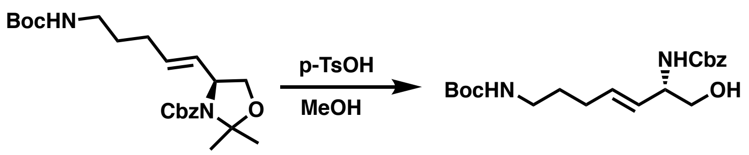

5 6

To a solution of compound **5** (1.21 g, 3 mmol, 1.0 equiv) in methanol (20 mL) was added p-toluenesulfonic acid (113 mg, 0.600 mmol, 0.2 equiv) and then the mixture was stirred overnight at room temperature. Methanol was evaporated under reduced pressure. The resulting crude product was purified by column chromatography using gradient elution (20% to 50% ethyl acetate in hexanes) to afford compound **6** as a colorless oil (0.98 g, 1.31 mmol, 87%).
Rf (1:1 EtOAc/hexanes): 0.32
^{1}H NMR (400 MHz, CDCl3): δ 7.31-7.42 (m, 5 H), 5.62-5.70 (m, 1 H), 5.50 (dd, J = 15.5, 5.5 Hz, 1 H), 5.07-5.18 (m, 3 H), 4.56-4.65 (m, 1 H), 4.22-4.30 (m, 1 H), 3.62-3.71 (m, 2 H), 3.12-3.23 (m, 2 H), 2.25-2.33 (br s, 1 H), 2.21 (m, 2 H), 1.43 (s, 9 H).
The ^{1}H spectral data match the literature precedent.[2]

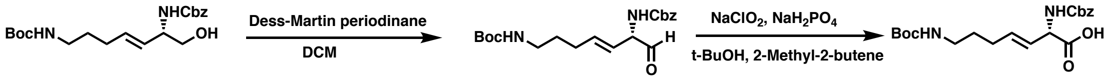

6 7

6 (726 mg, 1.92 mmol, 1 equiv.) was dissolved in dichloromethane (10 mL) in a 25 mL flame-dried flask under argon at 0 °C. Followed by adding Dess-Martin periodinane (1.62g, 3.83 mmol, 2 equiv.) and the mixture was stirred for 2 hours at 0°C. The reaction was filtrated over a plug of silica and the plug was washed with a mixture ethyl acetate/cyclohexane 1:1. Concentration to dryness afforded the air sensitive aldehyde which was used in the next step without further purification. The crude aldehyde was diluted in a mixture of tert-butanol / 2-methyl-2-butene (3:1, 40mL) and the resulting solution was cooled to 0 °C. A solution of sodium chlorite (1.96 g, 13.41 mmol, 7equiv.) and sodium phosphate monobasic (919.2 mg, 7.66 mmol, 4 equiv.) in water (10 mL) was slowly added and the reaction was stirred for 90 minutes at room temperature. The mixture was diluted with water and extracted with tert-butylmethyl ether. The combined organic layers were dried over Na_2SO_4, filtered and concentrated to dryness. The crude product was purified by flash column chromatography (0.2% acetic acid + 2% methanol in dichloromethane) to afford 306 mg (1.5 mmol, 78% over 2 steps) of **7** as a colorless solid.
Rf (0.5% acetic acid + 3% methanol in dichloromethane): 0.15
^{1}H NMR (400 MHz, CD3OD): δ 12.0 (br s, 1 H), 7.26-7.37 (m, 5 H), 5.76 (dt, J = 15.5, 7.2 Hz, 1 H), 5.63 (dd, J = 15.5, 6.0 Hz, 1 H), 5.09 (s, 2 H), 4.68 (d, J = 6.0 Hz, 1 H), 3.07 (t, J = 6.8 Hz, 2 H), 2.21 (dd, J = 13.5, 6.8 Hz, 2 H), 1.41 (s, 9 H).
The ^{1}H spectral data match the literature precedent.[2]

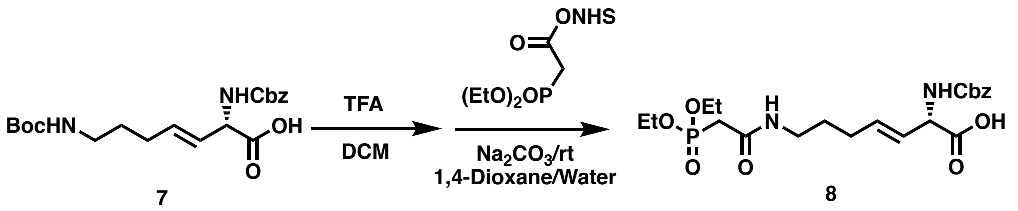

First, **7** (1513.7 mg, 4.0 mmol,1 equiv.) was dissolved in a 40% solution of TFA in DCM (8.0 mL, 4.0 mmol). The reaction was stirred for 45-60 minutes or until TLC indicated full consumption of starting material (TLC Conditions – DCM:MeOH, 10:1 v/v). The solution was then concentrated to dryness and used without purification. The material was then dissolved in 5 mL of a 1:1 Dioxane:Water mixture and was placed in an ice bath. To the reaction was added Na_2CO_3 (1695.8 mg, 16.0 mmol, 4 equiv.) and was stirred for 30 minutes at 0°C. Then, the phosphonohydroxysuccinate ester (1406.4mg 4.8 mmol,1.2 equiv.), as synthesized from literature, was dissolved in 2.5 mL of dioxane and added to the reaction, followed by 2.5 mL of water. The resulting mixture was stirred for 16 hours while warming to room temperature. Impurities from the reaction were then separated by extracting with ethyl acetate (2 x 50 mL). The aqueous layer was then acidified to pH = 2 with 1M HCl and then extracted again with ethyl acetate (3 x 50 mL). The organic layers were combined and washed with water (1 x 50 mL) and brine (1 x 50 mL). After drying and filtering, the organic layer was concentrated and resulting compound **8** used without further purification.

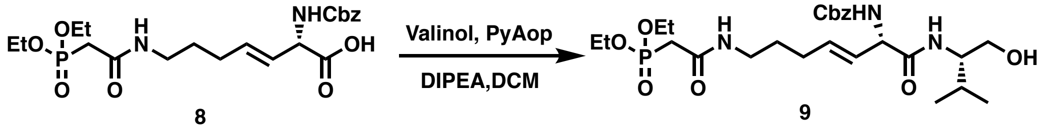

8 (232.8mg, 0.51 mmol, 1 equiv.) was dissolved in 5 mL of DCM and placed into an ice bath. PyAOP (318.5 mg, 0.61 mmol, 1.2 equiv.) was added to the mixture, followed by Valinol(58.0 mg, 0.56 mmol, 1.1 eq). After stirring for 5 minutes in the ice bath, DIPEA (133.2 µL, 0.77 mmol 1.5 equiv.) was slowly added before stirring at room temperature for 16 hours. Then, the reaction was evaporated and redissolved in 100 mL of ethyl acetate. The solution was then washed with 1 M HCl (3 x 50 mL), water (1 x 50 mL) and brine. The organic layer was collected, dried with sodium sulfate and evaporated before TLC and flash chromatography afforded **9** as a colorless oil. (211 mg, 0.38 mmol, 74%).

Rf (3:1 Acetone:DCM): 0.57

^{1}H NMR (400 MHz, $CDCl_3$): δ 7.46 – 7.30 (m, 5H), 6.86 (s, 1H), 6.72 (d, *J* = 8.8 Hz, 1H), 6.29 (s, 1H), 5.80 (dt, *J* = 14.5, 7.0 Hz, 1H), 5.65 (dd, *J* = 15.5, 7.0 Hz, 1H), 5.12 (d, *J* = 4.4 Hz, 2H), 4.71 (t, *J* = 6.4 Hz, 1H), 4.22 – 4.04 (m, 4H), 3.71 (tt, *J* = 17.1, 5.7 Hz, 3H), 3.55 (s, 1H), 3.36 (q, *J* = 5.9 Hz, 2H), 2.83 (d, *J* = 20.8 Hz, 2H), 2.30 (d, *J* = 6.9 Hz, 2H), 1.90 (dq, *J* = 13.7, 6.8 Hz, 1H), 1.34 (dt, *J* = 11.8, 7.1 Hz, 6H), 0.97 (dd, *J* = 11.3, 6.8 Hz, 6H).

The ^{1}H spectral data match the literature precedent.[3]

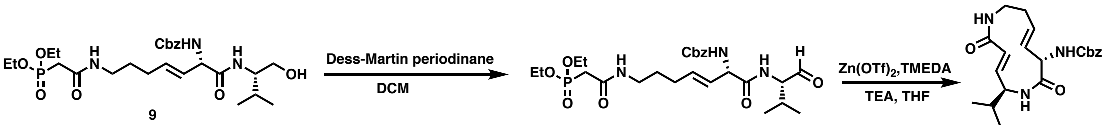

9 10

9 (108mg, 0.19 mmol, 1 equiv.) was dissolved in 2 mL of DCM and Dess Martin periodinane (90 mg, 0.21 mmol, 1.1 eq) was added. After stirring at room temperature for 90 minutes, the reaction was diluted in 100 mL of ethyl acetate. The organic solution was then washed with 1:1 mixture of saturated sodium bicarbonate and 2% $Na_2S_2O_3$ (2 x 50 mL). The organic phase was collected, dried, filtered and concentrated, which was used without further purification. TMEDA (35 µL, 0.23 mmol, 1.2 eq) and TEA (108 µL, 0.77 mmol, 4 eq) was added to a solution of $Zn(OTf)_2$ (155 mg, 0.42 mmol, 2.2 equiv.) in 60 mL of THF. The solution was stirred for 15-20 minutes before adding oxidized **9** in 40 mL of THF to a pressure-equalizing addition funnel. The solution in the funnel was added drop-wise to the reaction over 2 hours. The reaction was stirred for 20 hours at room temperature before concentrating and redissolving in 100 mL of ethyl acetate. Then, the solution was washed with 1 M HCl (1 x 50 mL), water (1 x 50 mL) and brine. Once the organic phase was collected, dried, filtered and concentrated, the resulting oil was crystalized in methanol and yield macrolactam **10** as colorless solid(24mg,0.063 mmol,30%)

Rf (1:1 Acetone:DCM): 0.38

^{1}H NMR (400 MHz, DMSO): δ 8.01 (d, *J* = 8.9 Hz, 1H), 7.51 (d, *J* = 7.6 Hz, 1H), 7.45 (t, *J* = 7.0 Hz, 1H), 7.42 – 7.26 (m, 5H), 6.69 (dd, *J* = 15.4, 5.5 Hz, 1H), 6.09 (d, *J* = 15.4 Hz, 1H), 5.63 (dt, *J* = 15.5, 7.6 Hz, 1H), 5.37 (dd, *J* = 15.8, 7.8 Hz, 1H), 5.09 – 4.96 (m, 2H), 4.66 (t, *J* = 7.7 Hz, 1H), 4.06 (dq, *J* = 17.3, 7.6 Hz, 1H), 3.26 – 3.05 (m, 2H), 2.33 – 2.24 (m, 1H), 1.99 – 1.87 (m, 1H), 1.75 (dd, *J* = 13.4, 6.7 Hz, 1H), 0.94 (dd, *J* = 16.5, 6.7 Hz, 6H).

The ^{1}H spectral data match the literature precedent.[3]

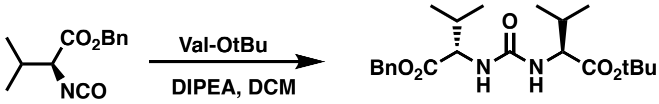

11 12

Isocyanate **11** (77.0 mg, 0.330 mmol, 1.0 equiv) was dissolved under nitrogen atmosphere in dichloromethane (5.0 mL) in a 25 mL flame-dried flask. A solution of L-valine tert-butyl ester (60.0 mg, 0.346 mmol, 1.05 equiv) and DIPEA (91.1 µL, 0.660 mmol, 2.0 eq) in dichloromethane (5.0 mL) was added and the resulting mixture was stirred overnight at room temperature. The reaction was quenched by addition of 10% aq. citric acid solution (5 mL) and the aqueous layer was extracted with dichloromethane (2×20 mL). The combined organic layers were washed with brine, dried (Na_2SO4), filtered and concentrated. The crude product was purified by column chromatography using gradient elution (10% to 30% ethyl acetate in hexane) to afford urea **12** (0.130 g, 0.320 mmol, 97%) as a colorless crystal.

Rf (1:2 EtOAc/hexanes): 0.62

^{1}H NMR (400 MHz, DMSO-d6): δ 7.32-7.40 (m, 5 H), 6.44 (d, J = 8.8 Hz, 1 H), 6.29 (d, J = 9.4 Hz, 1 H), 5.12 (ABq, J = 12.4 Hz, Δν= 22.6 Hz, 2 H), 4.11 (dd, J = 9.0, 5.3 Hz, 1 H), 3.94 (dd, J = 9.0, 5.3 Hz, 1 H), 1.96-2.05 (m, 2 H), 0.81-0.88 (m, 12 H).

The ^{1}H spectral data match the literature precedent.[2]

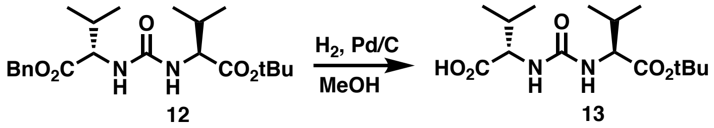

Urea **12** (41.0 mg, 0.101 mmol) was dissolved in methanol (8.0 mL) and then 10% Pd/C (4.0 mg) was added. The reaction was stirred under H_2 atmosphere for 60 minutes before filtration to remove the catalyst. The solvent was removed under reduced pressure. The resulting residue used without further purification.

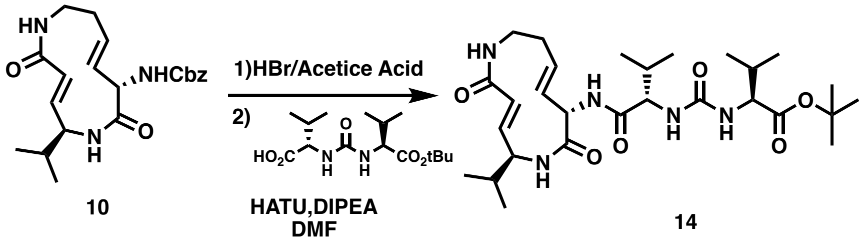

Macrocycle **10** (7.5 mg, 0.0195 mmol, 1.0 equiv) was dissolved in acetic acid (20 μL) followed by addition of hydrobromic acid (33 wt.% in acetic acid, 20 μL) under nitrogen atmosphere. The reaction mixture was stirred at room temperature for 30 minutes before evaporation in vacuo to dryness. The resulting orange powder was dissolved in DMF (0.2 mL) with N,N-diisopropylethylamine (6.8 μL, 0.0390 mmol, 2.0 equiv) at 0˚C, and add to a mixture of acid 13 (6.8 mg, 0.0215 mmol, 1.1 equiv), HATU (12.5 mg, 0.0292 mmol, 1.5 equiv) in DMF (0.8 mL) under nitrogen atmosphere. The reaction mixture was stirred overnight at room temperature before removing the solvent by evaporation. Acetone was added to the residue and separate the resulting precipitation from the suspension by centrifugation. The resulting solid was washed with acetone and acetonitrile, yielding compound **14** as a colorless solid. (5.5mg 0.01 mmol, 52%)

^{1}H NMR (400 MHz, DMSO-d6): δ 8.07 (d, J = 7.2 Hz, 1 H), 8.03 (d, J = 8.8 Hz, 1 H), 7.47 (t, J = 6.8 Hz, 1 H), 6.68 (dd, J = 15.5, 5.5 Hz, 1 H), 6.31 (d, J = 9.0 Hz, 1 H), 6.24 (d, J = 9.2 Hz, 1 H), 6.09 (d, J = 15.5 Hz, 1 H), 5.59 (dt, J = 15.5, 7.8 Hz, 1 H), 5.41 (dd, J = 15.8, 7.6 Hz, 1 H), 4.85 (m, 1 H), 4.02-4.12 (m, 2 H), 3.88 (dd, J = 8.8, 5.1 Hz, 1 H), 3.08- 3.25 (m, 2 H), 2.23-2.33 (m, 1 H), 1.86-2.02 (m, 3 H), 1.69-1.78 (m, 1 H), 0.94 (d, J = 6.9 Hz, 3 H), 0.90 (d, J = 6.9 Hz, 3 H), 0.82-0.87 (m, 9 H), 0.78 (d, J = 6.9 Hz, 3 H).

The ^{1}H spectral data match the literature precedent.[2]

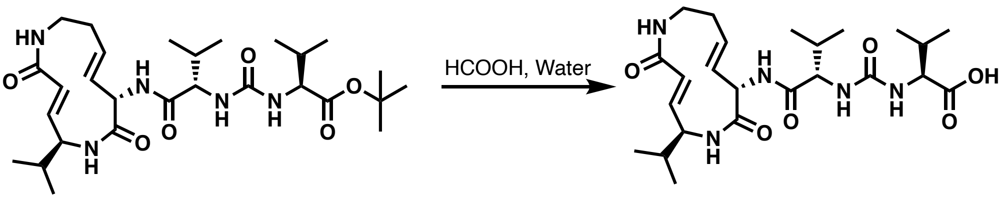

14 **Syringolin A**

Compound **14** (5.0 mg, 9.11 μmol) was dissolved in formic acid (0.4 ml) and the resulting mixture was stirred overnight at room temperature. After concentration to dryness, the crude product was washed with acetone and acetonitrile, yielding Syringolin A as a colorless solid.(4.4mg, 9.00 μmol, 98%)

^{1}H NMR (400 MHz, DMSO-d6): δ 12.5 (br s, 1 H), 7.98-8.06 (m, 2 H), 7.47 (t, J = 7.2 Hz, 1 H), 6.68 (dd, J = 15.5, 5.5 Hz, 1 H), 6.29 (d, J = 8.6 Hz, 1 H), 6.25 (d, J = 9.0 Hz, 1 H), 6.09 (d, J = 15.2 Hz, 1 H), 5.59 (dt, J = 15.8, 7.6 Hz, 1 H), 5.41 (dd, J = 15.6, 7.8 Hz, 1 H), 4.85 (m, 1 H), 4.02-4.12 (m, 2 H), 3.94 (dd, J = 8.8, 4.9 Hz, 1 H), 3.08- 3.25 (m, 2 H, overlapped with water), 2.23-2.32 (m, 1 H), 1.86-2.02 (m, 3 H), 1.68-1.78 (m, 1 H), 0.94 (d, J = 6.7 Hz, 3 H), 0.90 (d, J = 6.7 Hz, 3 H), 0.82-0.87 (m, 9 H), 0.77 (d, J = 6.9 Hz, 3 H);
The ^{1}H spectral data match the literature precedent.[2]

References

1. Garner, P.; Park, J. M., The synthesis and configurational stability of differentially protected. beta.-hydroxy-. alpha.-amino aldehydes. *The Journal of Organic Chemistry* **1987,** *52* (12), 2361-2364.
2. Dai, C.; Stephenson, C. R., Total synthesis of syringolin A. *Org Lett* **2010,** *12* (15), 3453-5.
3. Pirrung, M. C.; Biswas, G.; Ibarra-Rivera, T. R., Total synthesis of syringolin A and B. *Org Lett* **2010,** *12* (10), 2402-5.
