# Supplementary material for: Proteasomes accumulate in the plant apoplast where they participate in microbe-associated molecular pattern (MAMP)-triggered pathogen defense
Source: Nat Commun. 2025 Feb 14;16:1634. doi: 10.1038/s41467-025-56594-3 (PMC11829042; doi:10.1038/s41467-025-56594-3)
Supplement: Supplementary file 3 — Description of Additional Supplementary Files [file 41467_2025_56594_MOESM3_ESM.pdf]

Description of Additional Supplementary Files

Supplementary Data 1. Total list of 2,921 proteins (minus Rubisco) detected here by MS in the CL samples that match the APF.

Supplementary Data 2. Total list of 1,375 proteins (minus Rubisco) detected here by MS in the APF.

Supplementary Data 3. Total list of 219 proteins detected specifically by MS in the APF versus CL.

Supplementary Data 4. List of 29 proteins removed from the predicted apoplast list based on the know or predicted locations in other compartments

Supplementary Data 5. Curated list of 3,127 proteins predicted to be located in the apoplast.

Supplementary Data 6. Total list of 2,255 proteins (minus Rubisco) detected here by MS in the CL samples that match the AFPp.

Supplementary Data 7. Total list of 983 proteins (minus Rubisco) detected here by MS in the APFp.

Supplementary Data 8. Total list of 236 proteins detected specifically by MS in the APF versus CL.
