# Supplementary material for: Proteasomes accumulate in the plant apoplast where they participate in microbe-associated molecular pattern (MAMP)-triggered pathogen defense
Source: Nat Commun. 2025 Feb 14;16:1634. doi: 10.1038/s41467-025-56594-3 (PMC11829042; doi:10.1038/s41467-025-56594-3)
Supplement: Supplementary file 5 — Reporting Summary [file 41467_2025_56594_MOESM5_ESM.pdf]

nature portfolio

Corresponding author(s): Dr. Richard D. Vierstra

Last updated by author(s): Jan 10, 2025

Reporting Summary

Nature Portfolio wishes to improve the reproducibility of the work that we publish. This form provides structure for consistency and transparency in reporting. For further information on Nature Portfolio policies, see our Editorial Policies and the Editorial Policy Checklist.

Statistics

For all statistical analyses, confirm that the following items are present in the figure legend, table legend, main text, or Methods section.

n/a	Confirmed	
☐	☒	The exact sample size (*n*) for each experimental group/condition, given as a discrete number and unit of measurement
☐	☒	A statement on whether measurements were taken from distinct samples or whether the same sample was measured repeatedly
☐	☒	The statistical test(s) used AND whether they are one- or two-sided<br>*Only common tests should be described solely by name; describe more complex techniques in the Methods section.*
☒	☐	A description of all covariates tested
☐	☒	A description of any assumptions or corrections, such as tests of normality and adjustment for multiple comparisons
☐	☒	A full description of the statistical parameters including central tendency (e.g. means) or other basic estimates (e.g. regression coefficient) AND variation (e.g. standard deviation) or associated estimates of uncertainty (e.g. confidence intervals)
☐	☒	For null hypothesis testing, the test statistic (e.g. *F*, *t*, *r*) with confidence intervals, effect sizes, degrees of freedom and *P* value noted<br>*Give P values as exact values whenever suitable.*
☒	☐	For Bayesian analysis, information on the choice of priors and Markov chain Monte Carlo settings
☒	☐	For hierarchical and complex designs, identification of the appropriate level for tests and full reporting of outcomes
☒	☐	Estimates of effect sizes (e.g. Cohen's *d*, Pearson's *r*), indicating how they were calculated

Our web collection on statistics for biologists contains articles on many of the points above.

Software and code

Policy information about availability of computer code

Data collection	Thermo Fisher Xcalibur™ was used for proteomics data collection. CryoSPARC v4.2.1 was used for TEM image collection and processing. i-control 1.12 was used for fluorescence and absorbance data collection.
Data analysis	Proteome Discoverer 2.5 was used for proteomics data analysis. Microsoft Excel, Persus 2.0.11, GraphPad Prism 10 for statistical analysis and visualization.

For manuscripts utilizing custom algorithms or software that are central to the research but not yet described in published literature, software must be made available to editors and reviewers. We strongly encourage code deposition in a community repository (e.g. GitHub). See the Nature Portfolio guidelines for submitting code & software for further information.

Data

Policy information about availability of data

All manuscripts must include a data availability statement. This statement should provide the following information, where applicable:

- Accession codes, unique identifiers, or web links for publicly available datasets
- A description of any restrictions on data availability
- For clinical datasets or third party data, please ensure that the statement adheres to our policy

All data supporting the findings of this study are available in the article or its Supplementary Data, or from the corresponding author upon reasonable request. Source data are provided with this paper. The proteomic datasets are available at the Pride Repository under access code PXD059522.

Research involving human participants, their data, or biological material

Policy information about studies with human participants or human data. See also policy information about sex, gender (identity/presentation), and sexual orientation and race, ethnicity and racism.

Reporting on sex and gender	NA
Reporting on race, ethnicity, or other socially relevant groupings	NA
Population characteristics	NA
Recruitment	NA
Ethics oversight	NA

Note that full information on the approval of the study protocol must also be provided in the manuscript.

Field-specific reporting

Please select the one below that is the best fit for your research. If you are not sure, read the appropriate sections before making your selection.

☒ Life sciences ☐ Behavioural & social sciences ☐ Ecological, evolutionary & environmental sciences

For a reference copy of the document with all sections, see nature.com/documents/nr-reporting-summary-flat.pdf

Life sciences study design

All studies must disclose on these points even when the disclosure is negative.

Sample size	The sample size was considered based on our previous experiment and the feasibility of sample collection and analysis. The sample size for each experiment were indicated in figure legends.
Data exclusions	No data was excluded from the analyses.
Replication	Most experiments were conducted in two or more independent experiments with the number of biological replicates in each experiment specified in the figure legends, and all the independent experiments gave similar results.
Randomization	Plant materials were grown in the same conditions and the selection of plant materials was random.
Blinding	We did not apply blinding because of the experiments were carried out by a single person and blinding is generally not used for this kind of experiments.

Reporting for specific materials, systems and methods

We require information from authors about some types of materials, experimental systems and methods used in many studies. Here, indicate whether each material, system or method listed is relevant to your study. If you are not sure if a list item applies to your research, read the appropriate section before selecting a response.

Materials & experimental systems

n/a	Involved in the study	
☐	☒	Antibodies
☒	☐	Eukaryotic cell lines
☒	☐	Palaeontology and archaeology
☒	☐	Animals and other organisms
☒	☐	Clinical data
☒	☐	Dual use research of concern
☐	☒	Plants

Methods

n/a	Involved in the study	
☒	☐	ChIP-seq
☒	☐	Flow cytometry
☒	☐	MRI-based neuroimaging

Antibodies

Antibodies used	Anti-PAG1, anti-PBA1, anti-RPN1a, anti-RPN3, anti-RPN5, anti-PBF1, anti-rabbit Ub were prepared by the Vierstra whose use were previously reported. The dilution of above antibodies are 1:3000. Other antibodies were obtained commercially: anti-Rubisco large

subunit (Agrisera-AS03037), anti-cFBP (Agrisera-AS04043), anti-GFP (Abcam ab1218), anti-actin (Agrisera- AS132640), anti-FLAG (Millipore Sigma - F1804). Secondary antibodies were either the goat anti-mouse HRP conjugate (SeraCare, product number 0741806) or the goat anti-rabbit HRP conjugate (SeraCare, product number 0741506).

Validation

For proteasomal subunit antibodies, validation is based on the band size and pattern comparison with published papers.
Commercial antibodies were validated according to the information on the product website.
Agrisera : https://www.agrisera.com/
SeraCare : https://www.seracare.com/
Millipore Sigma : https://www.sigmaaldrich.com/US/en/product/sigma/f1804
srsltid=AfmBOop0ABGjUR467jChVUkCiz9hw4T8VV9ovCbNBINDjfUVKVfikvLx

Plants

Seed stocks

Arabidopsis wild-type Col-0 and Arabidopsis PAG1-FLAG transgenic line were reported in BooK et al., 2010, JBC. Arabidopsis atg5-1 and atg7-2 mutants were reported in Jeon et al., 2023, Autophagy. Arabidopsis atg12a atg12b mutant was reported in Chung et al., 2010, Plant J. Arabidopsis rpn10 mutant was reported in Marshall et al., 2015, Mol. Cell. The Arabidopsis atg5sid2 line was reported in Have et al., 2019, New Phytol.

Novel plant genotypes

No novel plant genotype is generated.

Authentication

PAG1-FLAG transgenic plant was validated by western blot with an anti-FLAG antibody.
Mutants were reported previously and tested by western blot or mass spectrometry-based proteomic analysis.
